# Bidirectional nucleolar dysfunction in *C9orf72* frontotemporal lobar degeneration

**DOI:** 10.1186/s40478-017-0432-x

**Published:** 2017-04-18

**Authors:** Sarah Mizielinska, Charlotte E. Ridler, Rubika Balendra, Annora Thoeng, Nathan S. Woodling, Friedrich A. Grässer, Vincent Plagnol, Tammaryn Lashley, Linda Partridge, Adrian M. Isaacs

**Affiliations:** 1 Department of Neurodegenerative Disease, UCL Institute of Neurology, Queen Square, London, WC1N 3BG UK; 2 Maurice Wohl Clinical Neuroscience Institute, King’s College London, Institute of Psychiatry, Psychology and Neuroscience, London, SE5 9RT UK; 3 Department of Genetics, Evolution and Environment, Institute of Healthy Ageing, University College London, Darwin Building, Gower Street, London, WC1E 6BT UK; 4 Institute of Virology, Saarland University Medical School, 66421 Hamburg, Germany; 5 UCL Genetics Institute, University College London, London, WC1E 6BT UK; 6 Department of Molecular Neuroscience, Queen Square Brain Bank, UCL Institute of Neurology, Queen Square, London, WC1N 3BG UK; 7 Max Planck Institute for Biology of Ageing, Joseph-Stelzmann-Strasse 9b, 50931 Cologne, Germany

**Keywords:** C9orf72, FTLD, Nucleolar stress, Dipeptide repeat proteins, Poly(GR), RNA foci

## Abstract

An intronic GGGGCC expansion in *C9orf72* is the most common known cause of both frontotemporal lobar degeneration (FTLD) and amyotrophic lateral sclerosis (ALS). The repeat expansion leads to the generation of sense and antisense repeat RNA aggregates and dipeptide repeat (DPR) proteins, generated by repeat-associated non-ATG translation. The arginine-rich DPR proteins poly(glycine-arginine or GR) and poly(proline-arginine or PR) are potently neurotoxic and can localise to the nucleolus when expressed in cells, resulting in enlarged nucleoli with disrupted functionality. Furthermore, GGGGCC repeat RNA can bind nucleolar proteins in vitro. However, the relevance of nucleolar stress is unclear, as the arginine-rich DPR proteins do not localise to the nucleolus in *C9orf72*-associated FTLD/ALS (C9FTLD/ALS) patient brain. We measured nucleolar size in C9FTLD frontal cortex neurons using a three-dimensional, volumetric approach. Intriguingly, we found that C9FTLD brain exhibited bidirectional nucleolar stress. C9FTLD neuronal nucleoli were significantly smaller than control neuronal nucleoli. However, within C9FTLD brains, neurons containing poly(GR) inclusions had significantly larger nucleolar volumes than neurons without poly(GR) inclusions. In addition, expression of poly(GR) in adult *Drosophila* neurons led to significantly enlarged nucleoli. A small but significant increase in nucleolar volume was also observed in C9FTLD frontal cortex neurons containing GGGGCC repeat-containing RNA foci. These data show that nucleolar abnormalities are a consistent feature of C9FTLD brain, but that diverse pathomechanisms are at play, involving both DPR protein and repeat RNA toxicity.

**Electronic supplementary material:**

The online version of this article (doi:10.1186/s40478-017-0432-x) contains supplementary material, which is available to authorized users.

## Introduction

An intronic GGGGCC expansion in *C9orf72* is the most common known cause of both frontotemporal lobar dementia (FTLD) and amyotrophic lateral sclerosis (ALS) [7, 27]. Healthy individuals have fewer than 30 repeats, whereas patients have several hundred to several thousand repeats [2, 7, 33]. The repeat expansion mutation might cause pathogenesis by loss of function of the C9orf72 protein, or gain-of-function mechanisms from i) sense and antisense repeat RNA and/or ii) the dipeptide repeat proteins poly(GA), poly(GP), poly(GR), poly(PR) and poly(AP), which are generated by repeat-associated non-ATG translation [28].

Previously, over-expression of poly(GR) and poly(PR) were reported to be extremely toxic to adult *Drosophila* neurons and primary rat neurons [19, 34]. Over-expression of poly(GR) or poly(PR) repeats in cell models leads to their localisation in the nucleolus, and results in enlarged nucleoli and altered ribosomal RNA processing [13, 32, 34]. Additionally, nucleolar proteins modify poly(PR) toxicity in yeast [12]. *C9orf72* repeat RNA has been shown to bind nucleolar proteins in vitro, suggesting that RNA toxicity may also contribute to nucleolar dysfunction [5, 10]. Dispersal of the nucleolar protein nucleolin was observed within neurons of adult *C9orf72* BAC transgenic mice, but no consequent change in ribosomal RNA biogenesis was detected [23]. However, enlarged nucleoli and altered ribosomal RNA processing have been reported in cells derived from patients with a *C9orf72* repeat expansion, including lymphocytes, fibroblasts and induced pluripotent stem cells differentiated into neurons [10]. Increases in nucleolar size and number are generally considered to be a consequence of cell demand for ribosome biogenesis, and are a hallmark of tumour cells in cancer [30]. However, disruption of nucleolar structure and ribosomal RNA transcription have also been reported in several neurodegenerative diseases, both in post-mortem patient tissue and animal models [25].

Recent proteomic studies have found that the binding partners of the arginine-rich DPR proteins are enriched in proteins containing low-complexity domains (LCDs), which are often found in membraneless organelles such as the nucleolus [14, 15]. The LCDs facilitate liquid-liquid phase separation, enabling cellular partitioning of membraneless organelles. The nucleolar protein nucleophosmin has an LCD that is bound by poly(GR) and poly(PR), altering its phase-separation properties and leading to altered nucleolar dynamics in cell culture assays [14]. These data suggest that disruption of the function of membraneless organelles is an important pathway in C9FTLD/ALS pathogenesis, and consequently confirmation of these findings in patient tissue is a key next step.

The relevance of nucleolar stress to disease pathogenesis has been questioned, as poly(GR) and poly(PR) inclusions do not localise to the nucleolus in C9FTLD/ALS patient brain and nucleolar size was reported to be unaffected in a small sample of C9FTLD/ALS brains [31]. To provide clarity to this important issue, we measured nucleolar size in C9FTLD brains using a three-dimensional, volumetric approach, rather than single-plane area measurements. We show here, for the first time, that nucleolar stress does occur in C9FTLD patient brain in a bidirectional manner and is associated with both repeat RNA and poly(GR) pathology.

## Materials and methods

### Human cases

Brain specimens (described in Additional file 1: Table S1) were obtained from Queen Square Brain Bank for Neurological Disorders, UCL Institute of Neurology, London. Samples were fixed in 10% buffered formalin for histopathology and immunohistochemistry. Histological sections from the anterior frontal F1-F2 region were analysed. We analysed eight controls with no known neurodegenerative disease, eight cases with heterozygous *C9orf72* repeat expansions, and one homozygous repeat expansion case. Seven expansion cases (cases 9, 12, 14–18) were previously described [20], including the homozygous repeat expansion case (case 17) [8]. The neuropathological diagnosis was determined using established diagnostic criteria, in line with consensus recommendations for the FTLD spectrum [16]. This study was approved by the UCL Institute of Neurology and National Hospital for Neurology and Neurosurgery Local Research Ethics Committee.

### Fly stocks and husbandry

All fly stocks were maintained and experiments conducted at 25 °C on a 12 h:12 h light:dark cycle at 60% constant humidity, on standard sugar-yeast food containing 15 g/L agar, 50 g/L sugar, 100 g/L brewer’s yeast, 30 ml/L nipagin (10% in ethanol) and 3 ml/L propionic acid). RU486 (Sigma) dissolved in ethanol was added to a final concentration of 200 μM. Published transgenic fly lines expressing 100 repeats of the DPR proteins GR and glycine-alanine (GA), under the upstream activating sequence (UAS) promoter, were used [19]. Expression of these DPR proteins was restricted to adult neurons using the inducible elav-GeneSwitch driver [24]. Two days after eclosion adult elav-GeneSwitch > (GR)100 and elav-GeneSwitch > (GA)100 flies were fed with food containing 200 μM RU486 to induce expression of the DPR proteins, or with no RU486 as a control, for 7 days.

### Immunofluorescence

#### Human post-mortem brain

Twenty micrometer-thick paraffin-embedded sections of human post-mortem frontal cortex tissue were dewaxed in xylene and rehydrated in graded alcohols, followed by antigen-retrieval in proteinase K (S3020, Dako) for 2 mins, and pressure cooking in citrate buffer (0.1 M, pH6) for 10 mins. Following a PBS wash, sections were blocked in 10% foetal bovine serum in PBS for 30 mins, and then incubated with primary antibody in PBS overnight at 4 °C. The following primary antibodies were used: nucleophosmin (1:1000, Abcam, ab10530), nucleolin (1:50, Santa Cruz, sc-8031), NeuN (1:1000, Millipore, ABN78), and 5H9 antibody against poly(GR) (1:25, [21]). Samples were then washed in PBS and incubated with the appropriate secondary antibodies (Alexa Fluor, Life Technologies, 1:500 in PBS) for 1 h at room temperature. To reduce autofluorescent background, a 10 min incubation was carried out in Sudan black B (Sigma, 0.2% in 70% ethanol/30% PBS) followed by additional PBS washes. Samples were mounted using ProLong® Gold Antifade Mountant with DAPI (Life Technologies).

#### Drosophila

Brains were dissected in PBS containing 0.3% Triton-X (PBST) and then fixed in 4% PFA in PBST at room temperature for 15 min. Brains were washed three times in PBST, blocked in 10% bovine serum albumin (BSA) in PBST for 1 h at room temperature, and then incubated in primary antibody in 10% BSA in PBST at 4 °C for 48 h. The following primary antibodies were used: poly(GR) (1:25, 5H9 - as above), poly(GA) (1:300, Cosmobio, CAC-TIP-C9-P01), fibrillarin (1:400, Abcam, ab4566). Brains were washed in PBST and incubated with appropriate secondary antibodies (Alexa Fluor, Life Technologies, 1:250 in 10% BSA in PBST) at 4 °C overnight. Brains were then washed three times in PBST and whole mounted onto glass slides in VECTASHIELD® mounting medium with DAPI (Vector Laboratories, H-1200).

### RNA fluorescent in situ hybridisation with immunofluorescence

Twenty micrometer-thick paraffin-embedded sections of human post-mortem frontal cortex tissue were dewaxed followed by antigen-retrieval as above. The protocol was then continued as per Mizielinska et al., 2013 [20]. Briefly, sections were washed in 2 × SSC and incubated for 30 min in pre-hybridisation solution (50% formamide/2 × SSC) at 80 °C, and hybridised with a (GGCCCC)_4_ 2′-O-methyl RNA probe labelled with Cy3 (Integrated DNA Technologies) for 2 h at 80 °C in hybridisation solution (50% formamide, 2 × SSC, 0.8 mg/ml tRNA, 0.8 mg/ml salmon sperm DNA, 0.16% BSA, 8% dextran sulphate, 1.6 mM ribonucleoside vanadyl complex, 5 mM EDTA, 0.2 ng/μl probe). Sections were washed three times for 30 min each in 50% formamide/0.5 × SSC at 80 °C, and then three times for 10 min at room temperature in 0.5 × SSC. After a brief wash in PBS, immunostaining was continued from blocking as above.

### Image acquisition and quantification of nucleolar volume

Images of human post-mortem brain were acquired using an LSM710 confocal microscope (Zeiss) using a plan-apochromat 40×/1.4 NA oil immersion objective. Fluorescence intensity was set to peak for each patient to account for case-to-case variability. Images of *Drosophila* brains were acquired with an LSM700 confocal microscope (Zeiss) using a plan-apochromat 63× oil/1.4 NA immersion objective and the same settings for all images.

#### Immunofluorescence of human post-mortem brain

Twenty z-stack images were acquired per sample, consisting of twelve 2 μm z-planes of 2084 × 2084 pixels, over a 20 μm depth (of which approximately 14 μm contained tissue after processing). A minimum of 200 neurons were analysed (range 217–973), including a minimum of 16 poly(GR) aggregate-bearing neurons (range 16–139) per C9FTLD frontal cortex. For detection of nuclei, nucleoli and NeuN, gain was adjusted to peak intensity for each patient. Three-dimensional volumetric analysis of confocal images was performed using Volocity image analysis software (Perkin Elmer). Nucleoli (identified by either nucleophosmin or nucleolin), DAPI-stained nuclei and NeuN-positive neurons were identified by fluorescence-intensity threshold a set number of standard deviations above mean voxel intensity for each image. As poly(GR) aggregates are present in C9FTLD patient sections but not control sections, an absolute intensity threshold was used for detection. Low-level poly(GR) signal was observed inside the nucleolus in both cases and controls and was consequently excluded as background. No intra-nucleolar poly(GR) aggregates were observed by eye. Objects defined as nucleoli, poly(GR) aggregates and nuclei were compartmentalised into NeuN-positive neurons to give the volume of each stain present in each neuron. Occasionally neurons contained more than one nucleoli; the volumes of these structures were combined for analysis. Neurons in which no nucleolar stain was detected were excluded from analysis.

#### Immunofluorescence of Drosophila

Four z-stack images covering a 15 μm depth were taken from each *Drosophila* brain, achieving a minimum of 174 nucleoli per brain (range 174–920), including a minimum of five poly(GR) (range 5–63) or eight poly(GA) aggregates (range 8–53). Image acquisition and volumetric analysis of DAPI-stained nuclei, nucleoli and poly(GR) or poly(GA) aggregates was performed similarly to human post-mortem brain, except that no exclusion of background nucleolar staining of aggregates was required and aggregates were assigned to nucleoli by proximity of the centroids. Occasional low-level staining of poly(GR) or poly(GA) was observed in uninduced flies owing to the known leaky expression of the elav-GeneSwitch driver [26]; nucleoli associated with these aggregates were excluded from the subsequent analysis.

#### RNA fluorescent in situ hybridisation with immunofluorescence in human post-mortem brain

Ten to seventeen z-stack images at 2084 × 2084 pixels covering a 20 μm depth were acquired to ensure a minimum of 100 neurons were analysed (range 134–232), including a minimum of 38 RNA foci-bearing neurons (range 38–88) per C9FTLD frontal cortex. Image acquisition and volumetric analysis of nucleoli and poly(GR) aggregates was performed similarly to human post-mortem brain immunofluorescence analysis described above; RNA foci were additionally counted using the touch count tool; neurons were determined either by NeuN-positive staining or by morphology (size and DAPI staining).

#### Statistical analysis

Data are presented as median nucleolar or nuclear volume per individual patient or fly owing to the non-normal distribution of volumes in all data sets. Unpaired *t* tests were carried out to compare median nucleolar and nuclear volumes between control and C9FTLD patient brain, and between uninduced and induced (GR)100 or (GA)100 transgenic *Drosophila*. Paired regression analysis was carried out between neurons with and without pathology (DPR protein inclusions or RNA foci) in C9FTLD patient brain or *Drosophila*.

## Results

### Nucleolar volume is reduced in C9FTLD patient brain

**Fig. 1:**
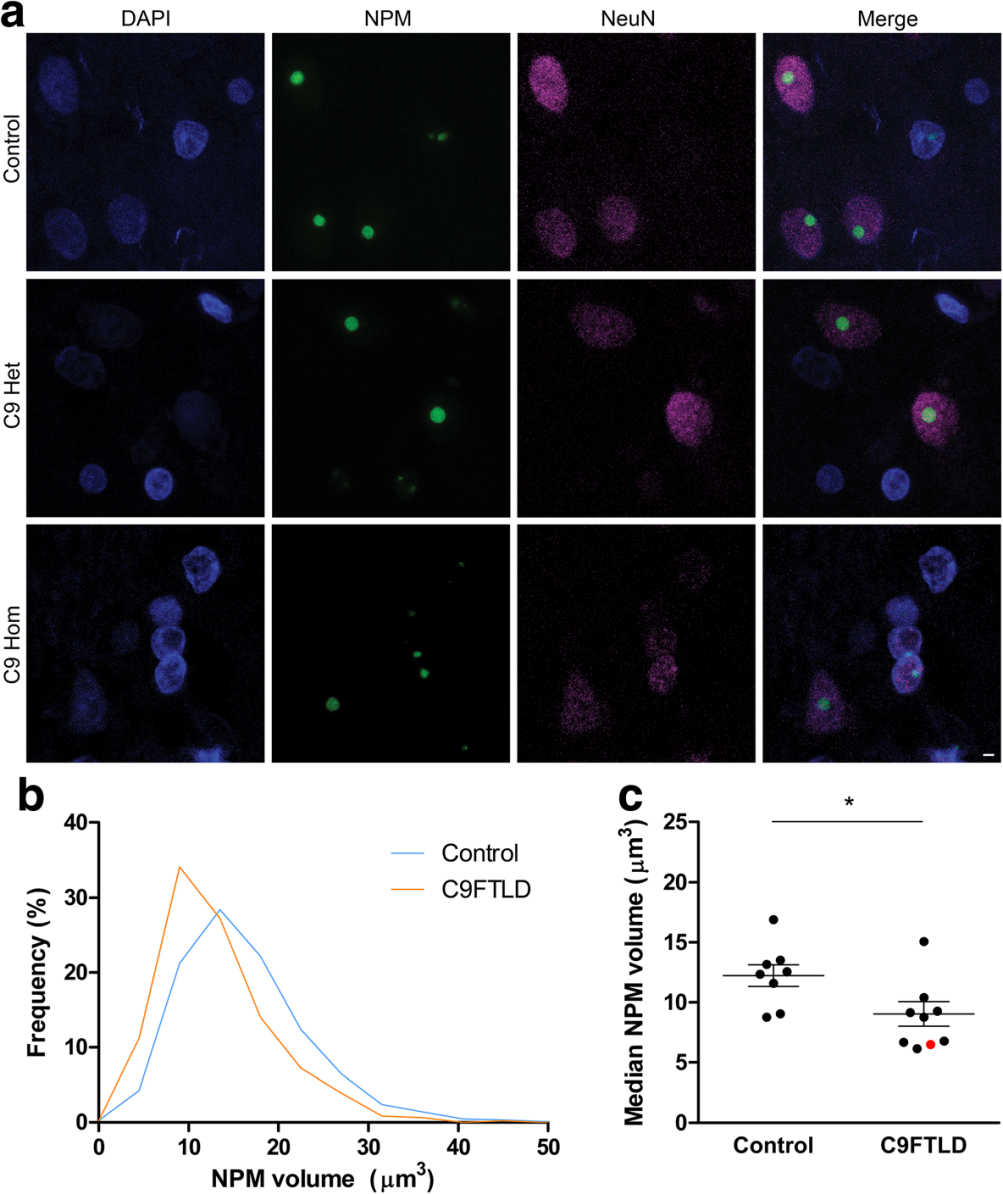
Decreased nucleolar volume in neurons from C9FTLD patient brain compared with neurons from neurologically-normal control brain. **a** Representative images of frontal cortex from neurologically-normal controls and heterozygous (C9 Het) and homozygous (C9 Hom) C9FTLD cases immunostained for the nucleolar protein nucleophosmin (NPM, *green*), the neuronal marker (NeuN, *magenta*) with DAPI nuclear stain (*blue*). *Scale bar* represents 2 μm. **b**, **c** Quantification of neuronal nucleolar volume determined by nucleophosmin immunoreactivity. Frequency distribution of pooled control and C9FTLD (heterozygous cases only) nucleolar volumes show a shift to reduced volumes in C9FTLD cases (**b**). Median nucleolar volume was significantly decreased in C9FTLD cases compared with controls (**c**). Each *dot* represents an individual case with the homozygous C9FTLD case shown in *red*, and the average and SEM of heterozygous cases shown as *long* and *short horizontal bars*, respectively. Significance was determined by unpaired *t* test: *p < 0.05

To determine whether nucleolar volume was altered in C9FTLD patient brain, we used confocal microscopy to capture three-dimensional z-stack images from sections of frontal cortex in eight heterozygous and one homozygous C9FTLD case, and eight non-neurodegenerative disease controls (details of cases in Additional file 1: Table S1). These sections were stained for two nucleolar markers (nucleophosmin and nucleolin) the neuronal marker NeuN. Both control and patient brain displayed clear nucleophosmin-positive nucleoli in neurons (Fig. 1a). Analysis of at least 200 neurons per case showed no difference in the number of nucleophosmin-positive nucleoli between controls and C9FTLD cases, with most cells containing only one nucleolar structure (Additional file 1: Figure S1a). Plotting a frequency distribution of total nucleophosmin volume per neuron showed a broad distribution in both control and C9FTLD patient frontal cortex, but with C9FTLD patient neurons showing a shift towards reduced volumes of nucleophosmin per cell (Fig. 1b). Analysis of median nucleophosmin volume showed that C9FTLD cases had on average 26% smaller nucleoli in frontal cortical neurons than control cases (9.0 ± 1.0 versus 12.2 ± 0.9 μm^3^, *p* < 0.05, Fig. 1c). Median nucleophosmin volume in the single homozygous C9FTLD case was at the bottom of the range observed for heterozygous cases. Importantly, there was no difference in median nuclear volume between controls and C9FTLD cases (Additional file 1: Figure S1b), indicating that the observed change in nucleolar volume was not simply due to enlarged nuclei. Nucleophosmin volume in neurons showed a weak correlation with nuclear volume (Additional file 1: Figure S1c and d), therefore nucleolar volumes were not corrected for nuclear volume. Analysis of the distinct nucleolar marker nucleolin showed that median nucleolin volume was lower than nucleophosmin volume, with no difference in the number or volume of nucleolin-positive structures identified (Additional file 1: Figure S2). These data show that the nucleophosmin-positive nucleolar volume is decreased in neurons from C9FTLD frontal cortex compared with controls.

### Nucleolar volume is increased in poly(GR) inclusion-bearing neurons in C9FTLD patient brain

**Fig. 2:**
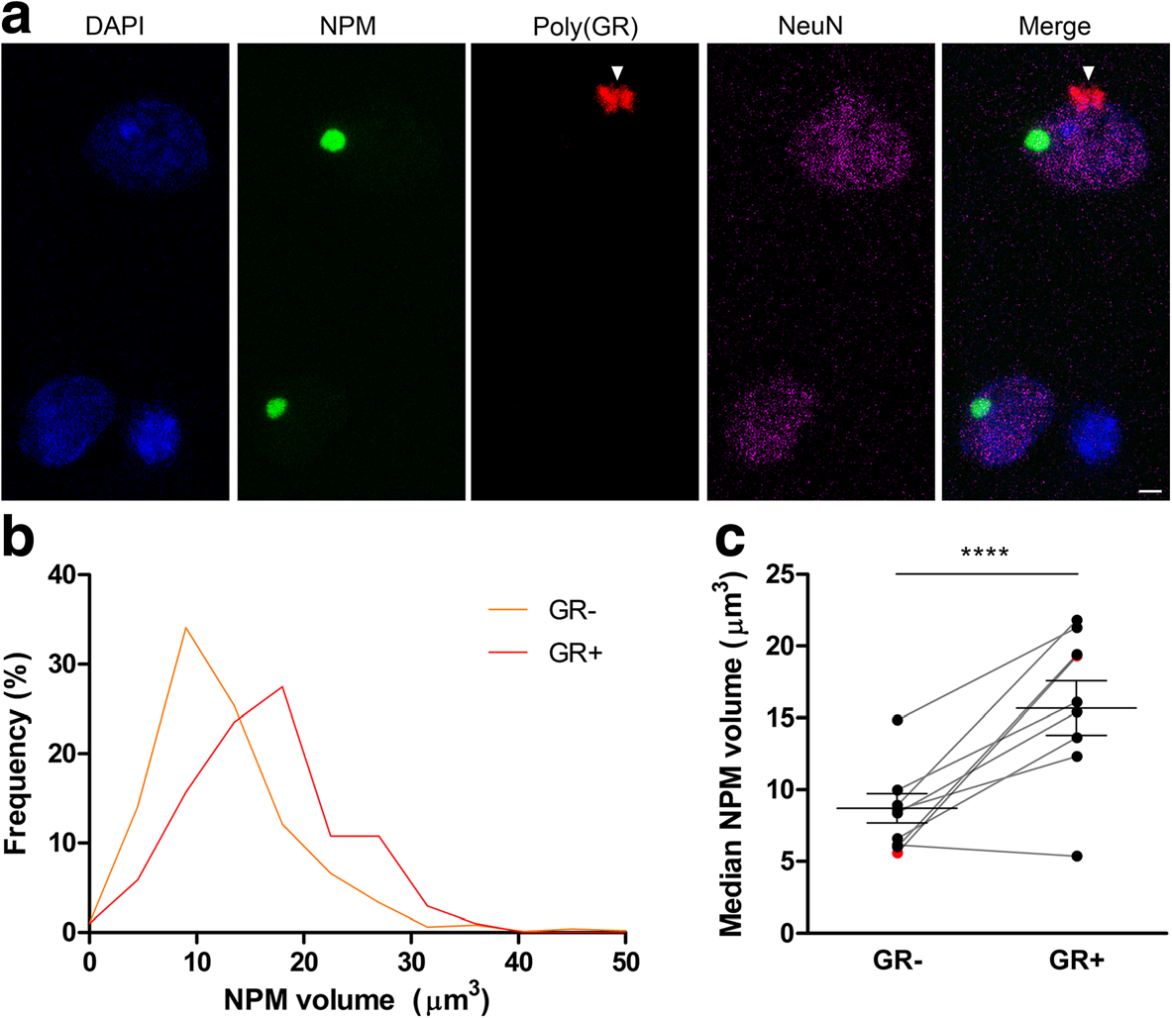
Increased nucleolar volume in poly(GR) inclusion-bearing neurons in C9FTLD patient brain. **a** Representative images of frontal cortex from a heterozygous C9FTLD case immunostained for the nucleolar protein nucleophosmin (NPM, *green*), poly(GR) protein (*red*), the neuronal marker (NeuN, *magenta*) with DAPI nuclear stain (*blue*); a typical poly(GR) inclusion is *arrowed. Scale bar* represents 2 μm. **b**, **c** Quantification of neuronal nucleolar volume determined by nucleophosmin immunoreactivity. Frequency distribution of pooled C9FTLD (heterozygous cases only) nucleolar volumes show a shift to increased volumes in neurons bearing poly(GR) inclusions compared with neurons without inclusions (**b**). Median nucleolar volume in C9FTLD cases was significantly larger in neurons with poly(GR) inclusions (GR+) compared with neurons without inclusions (GR-) (**c**). Each *dot* represents an individual case with the homozygous C9FTLD case shown in *red*, *grey lines* link medians from the same cases in neurons with or without poly(GR) inclusions, and the average and SEM of heterozygous cases are shown as *long and short horizontal bars*, respectively. Significance was determined by paired regression analysis: ****p < 0.0001

We next investigated whether nucleolar volume was altered in the presence of C9FTLD-specific pathologies. The data for C9FTLD nucleolar volume were segregated into poly(GR) inclusion-bearing and non-inclusion bearing neurons, as the sections were also immunostained for poly(GR) protein (Fig. 2a). We analysed images that contained both GR-positive and -negative neurons to ensure the laminar distribution of each population was matched. Only cytoplasmic poly(GR) inclusions were assessed as nuclear inclusions were too infrequent for analysis. In these data, C9FTLD frontal cortical neurons bearing a poly(GR) inclusion showed no change in the number of nucleophosmin-positive nucleoli per cell compared to neurons without an inclusion (Additional file 1: Figure S3a), but a strong shift towards increased nucleophosmin volumes (Fig. 2b). Analysis of median nucleophosmin volume per case showed that C9FTLD poly(GR) inclusion-bearing neurons had 87% bigger nucleoli on average than neurons without an inclusion (15.7 ± 1.9 versus 8.7 ± 1.0 μm^3^, *p* < 0.0001, Fig. 2c). Median nucleophosmin volume in the single homozygous C9FTLD case was not notably different from heterozygous cases. No differences were detected in nuclear volume between neurons with or without inclusions (Additional file 1: Figure S3b). There was no change in the number of nucleolin structures per nuclei, but median nucleolin volume was increased on average by 74% in poly(GR) inclusion-bearing neurons in C9FTLD cases compared to neurons without inclusions (9.1 ± 0.9 versus 5.2 ± 0.5 μm^3^, *p* < 0.001, Additional file 1: Figure S4). Notably, cytoplasmic poly(GR) inclusions were present in 6.2 ± 1.3 and 6.4 ± 1.3% of neurons in nucleophosmin and nucleolin datasets, respectively. These data show that nucleolar volume is considerably enlarged in C9FTLD neurons that contain poly(GR) inclusions compared with those without.

### Nucleolar enlargement can be evoked by expression of poly(GR) protein in vivo

**Fig. 3:**
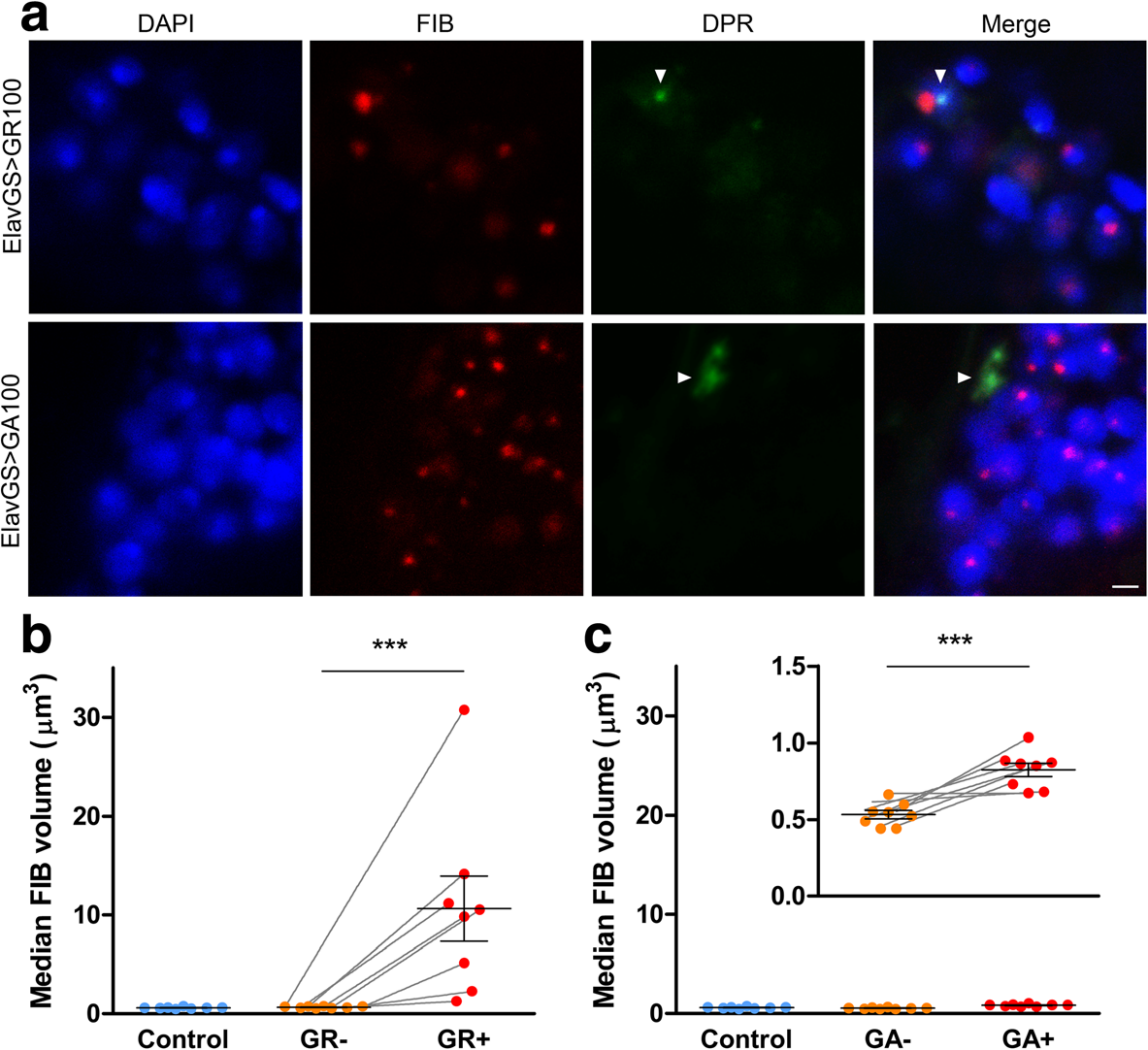
Expression of GR100, and to a lesser extent GA100, in *Drosophila* adult neurons is sufficient to increase nucleolar volume in inclusion-bearing neurons. **a** Representative images of *Drosophila* brain expressing GR100 or GA100 in adult neurons using the elav-GeneSwitch (elavGS) driver immunostained for the nucleolar protein fibrillarin (FIB, *red*), the dipeptide repeat (DPR) protein poly(GR) or poly(GA) (*green*), with DAPI nuclear stain (*blue*); typical poly(GR) and poly(GA) inclusions are arrowed. Scale bar represents 2 μm. **b**, **c** Quantification of neuronal nucleolar volume determined by fibrillarin immunoreactivity. Median nucleolar volume in *Drosophila* adult neurons expressing GR100 was significantly larger in neurons bearing poly(GR) inclusions (GR+) than in neurons without inclusions (GR-) (**b**). Using the same scale as in **b**, the median nucleolar volume increase in *Drosophila* adult neurons expressing GA100 is not apparent, highlighting the difference in magnitude of the changes; magnifying the scale (*inset*), reveals nucleolar volume was significantly larger in neurons bearing poly(GA) inclusions (GA+) than in neurons without inclusions (GA-) (**c**). Controls in **b** and **c** are *Drosophila* transgenic for DPR proteins that were not induced for gene expression. Each dot represents an individual fly, grey lines link medians from the same individual fly in neurons with or without DPR protein inclusions, and average and SEM are shown as *long and short horizontal bars*, respectively. Significance was determined by paired regression analysis: ***p < 0.001. Genotypes were: w; UAS-GR100/+; elavGS/+ (elavGS > GR100), w; UAS-GA100/+; elavGS/+ (elavGS > GA100)

As pathologies from the five distinct DPR proteins at least partially overlap in patient tissue [21], we investigated the potential for individual DPR proteins to exert changes in nucleolar morphology in isolation, but still within an in vivo system. We analysed *Drosophila* lines expressing either 100 repeats of poly(GR) or of poly(GA) in adult neurons, both of which we have previously shown to display neurotoxicity [19]. Inclusions of both poly(GA) and poly(GR) were found to be widely distributed throughout *Drosophila* brains (Fig 3a), being present in 7.8 ± 2.3 and 6.6 ± 1.9% of neurons, respectively (Additional file 1: Figure S5). Neuronal nucleoli associated with poly(GR) inclusions were about 18-fold larger on average than nucleoli without inclusions (10.6 ± 3.3 versus 0.60 ± 0.03 μm^3^, *p* < 0.001), which were no different to nucleoli in control flies (Fig. 3b). Neuronal nucleoli associated with poly(GA) inclusions in GA(100) flies were 1.5-fold larger than nucleoli without inclusions (0.83 ± 0.04 versus 0.53 ± 0.03 μm^3^, *p* < 0.001), which were again the same size as nucleoli in control flies (Fig. 3c). These findings show that the arginine-rich DPR protein poly(GR) can exert nucleolar stress to a far greater extent than poly(GA) in vivo.

### Nucleolar volume is increased in RNA foci-bearing neurons in C9FTLD patient brain

**Fig. 4:**
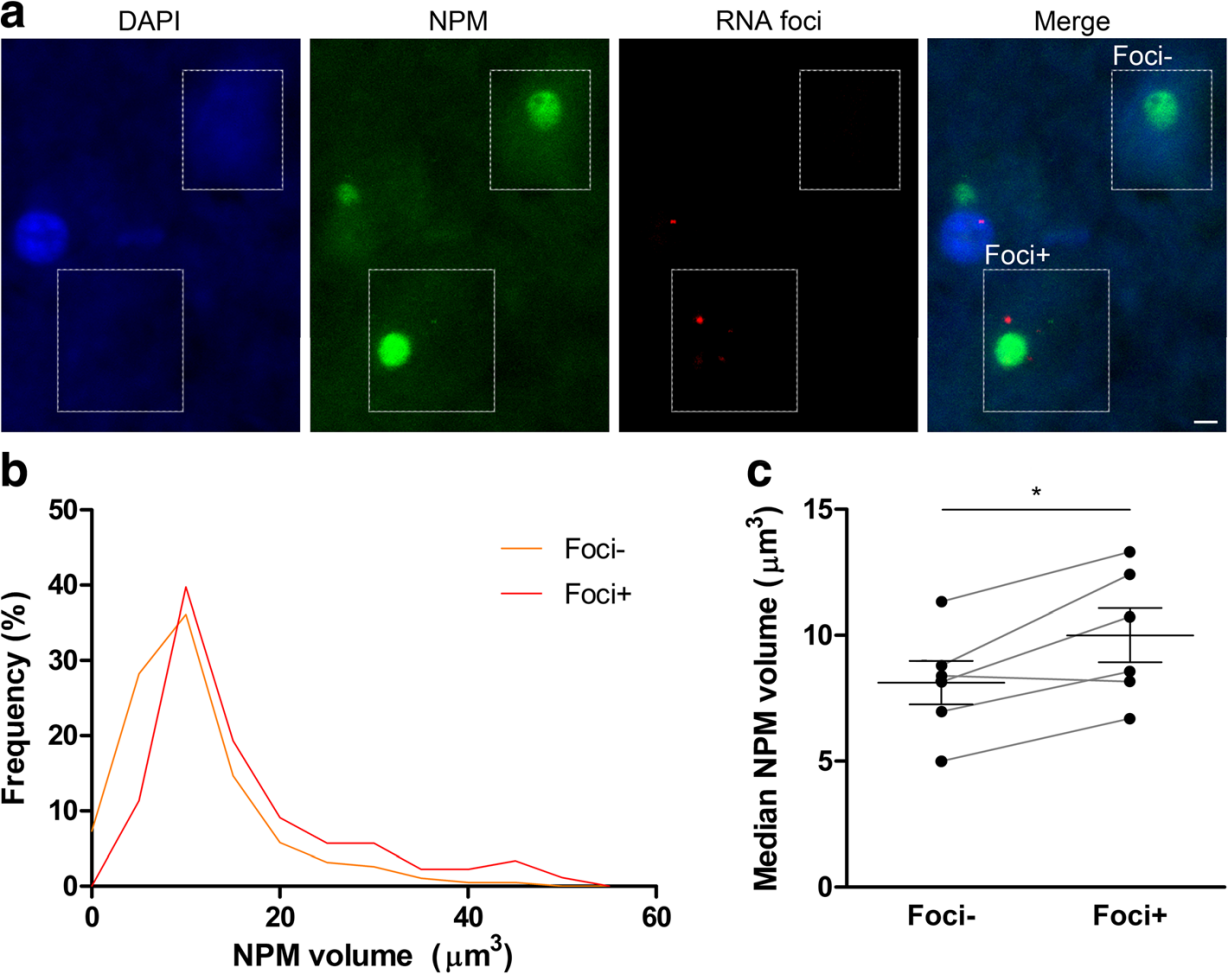
Nucleolar volume is increased in RNA foci-bearing neurons in C9FTLD patient brain. **a** Representative images of frontal cortex from a heterozygous C9FTLD case immunostained for the nucleolar protein nucleophosmin (NPM, *green*), with RNA fluorescent in situ hybridisation for sense RNA foci (*red*) and DAPI nuclear stain (*blue*); typical RNA foci-bearing (Foci+) and non foci-bearing (Foci-) neurons are highlighted with dotted boxes. Scale bar represents 2 μm. **b**, **c** Quantification of neuronal nucleolar volume determined by nucleophosmin immunoreactivity. Frequency distribution of pooled C9FTLD (heterozygous cases) nucleolar volumes show a slight shift to increased volumes in neurons bearing RNA foci compared with neurons without foci (**b**). Median nucleolar volume in C9FTLD cases was significantly larger in neurons with RNA foci compared with neurons without foci inclusions (**c**). Each dot represents an individual heterozygous C9FTLD case, grey lines link medians from the same cases in neurons with or without RNA foci, and the average and SEM are shown as *long and short horizontal bars*, respectively. Significance was determined by paired regression analysis: *p < 0.05

In addition to reports of association between the arginine-rich DPR proteins and the nucleolus, *C9orf72* sense repeat RNA has also been shown to bind nucleolar proteins in vitro [5, 10]. To investigate whether any changes in nucleolar volume were associated with RNA foci in patient tissue, we performed fluorescent in situ hybridisation for *C9orf72* sense RNA combined with immunofluorescence for the nucleolar marker nucleophosmin on C9FTLD patient frontal cortex (Fig. 4a). Tissue was also stained for poly(GR) protein, and neurons containing poly(GR) inclusions were excluded from the analysis (Additional file 1: Figure S6a–c). RNA foci were present in an average of 35.0 ± 4.3% of neurons (Additional file 1: Figure S6b). C9FTLD frontal cortical neurons bearing sense RNA foci showed no difference in the number of nucleophosmin-positive nucleoli per cell (Additional file 1: Figure S6d), but a small shift towards increased nucleophosmin volume compared with neurons without foci (Fig. 4b). Analysis of median nucleophosmin volume per case showed that RNA foci-bearing neurons had on average 24% bigger nucleoli than neurons without foci (10.0 ± 1.1 versus 8.1 ± 0.9 μm^3^, *p* < 0.05, Fig. 4c). We were not able to determine nuclear size as DAPI staining was not robust enough to enable volume analysis after our combined FISH and immunostaining protocol (FISH at 80 °C and protease antigen retrieval). However, reanalysing a previous data set using four of the six cases described here [20] showed no difference in nuclear volume between neurons with or without RNA foci (Additional file 1: Figure S6e). Interestingly, an average of 18.4 ± 1.4% of RNA foci were observed to colocalise with nucleophosmin, mostly on the edge of the nucleolar structure (Additional file 1: Figure S7a, b). In fact, in RNA foci-bearing neurons, nucleoli directly associated with foci had a 26% larger nucleophosmin volume than neurons where foci were present only in the nucleoplasm (11.8 ± 1.5 versus 9.3 ± 1.0 μm^3^, *p* < 0.01, Additional file 1: Figure S7c). This finding suggests that RNA foci induce nucleolar enlargement predominantly through direct interaction with the nucleophosmin component of nucleoli.

## Discussion

Here, we present evidence for *C9orf72* mutation-associated nucleolar alterations in patient brain. Using three-dimensional volumetric imaging, we have unveiled bidirectional changes in nucleolar size in C9FTLD patient brain. When comparing all frontal cortical neurons irrespective of the presence of RNA or protein pathology, C9FTLD cells had a smaller nucleophosmin-positive nucleolar volume than those in non-neurodegenerative control brains. However, within C9FTLD neurons, the presence of poly(GR) inclusions or RNA foci resulted in significant enlargement of nucleoli.

Thus far striking in vitro evidence exists for increased nucleolar size in both cells overexpressing the arginine-rich DPR proteins [13, 32, 34], and cells derived from C9FTLD/ALS patients [10]. However, it is perhaps unsurprising that neurons in C9FTLD frontal cortex displayed an overall reduction in nucleolar size, as this type of nucleolar stress has been observed in several other neurodegenerative diseases [25]. Nucleolar shrinkage would be expected to reduce ribosome biogenesis and generally decrease cell metabolism. Indeed, reduced ribosomal RNA maturation has been reported in C9ALS patient lymphocytes and motor cortex [10]. Acutely, this change might be protective under conditions of cell stress by limiting energy expenditure, but chronically would result in cell damage and ultimately cell death [9]. The nucleolar protein nucleophosmin is known to be downregulated upon excitotoxic stimuli in neurons and can increase levels of the classic nucleolar stress marker p53; however, nucleophosmin-induced cell death appears to be p53-independent [17].

In agreement with previous in vitro evidence (detailed above), we also unveiled a second type of nucleolar stress in C9FTLD neurons containing inclusions of the arginine-rich DPR protein poly(GR), in which nucleolar volume was almost double that of neurons without inclusions. This increased volume could be expected to increase ribosomal biogenesis; however, initial studies in vitro show reduced ribosomal RNA production when the arginine-rich DPR proteins are overexpressed in cells [13, 32]. These findings could suggest that the enlarged nucleoli are dysfunctional rather than implying an increased metabolism. Increases in nucleophosmin-positive nucleolar size owing to the presence of poly(GR) inclusions were replicated with another nucleolar protein nucleolin, although no difference was detected between control and C9FTLD cases. Both nucleophosmin and nucleolin localise to the nucleolus, but nucleophosmin is predominantly found in the granular centre of nucleoli, and nucleolin in both the dense fibrillary centre and the granular centre of nucleoli. Volume measurements for nucleolin were lower than those for nucleophosmin, which might reflect differences in detection sensitivity. Further work using super-resolution microscopy would be required to investigate these sub-nucleolar structures in more detail.

One discrepancy between cell models of C9FTLD/ALS and patient tissue is the localisation of the poly(GR) protein inclusions. In cells, short poly(GR) peptides readily localise to the nucleolus where they exert the aforementioned nucleolar enlargement and impaired ribosome biogenesis [13]. By contrast, in patient brain, poly(GR) protein is detected primarily in large cytoplasmic inclusions and occasionally in small dot-like perinucleolar inclusions [1, 22]. Here, we have provided evidence linking cytoplasmic poly(GR) inclusions in patient tissue to changes in nucleolar volume, which has previously only been detected in cell models. Although poly(GR) protein is not detected in the nucleolus in C9FTLD patient brain, cytoplasmic inclusions might be present primarily in neurons that have a high poly(GR) protein load, including soluble protein, undetectable by the immunofluorescence methods used in this study.

It is likely that a proportion of the cytoplasmic inclusions that are detected with poly(GR) immunostaining also contain some of the other dipeptide repeat proteins, as the DPR proteins have previously been shown to co-occur within individual inclusions [21]. The most commonly occurring DPR protein pathology is inclusions containing poly(GA) protein, which also evoke neurotoxicity [18, 19, 35, 36]. However, we specifically investigated the effect of poly(GR) and poly(GA) proteins on neuronal nucleoli in vivo, and observed a much higher capability for poly(GR) to evoke these changes than poly(GA) protein. Poly(GR) protein is thus highly likely to be responsible for the nucleolar enlargement associated with the poly(GR) inclusion pathology that we have detected in C9FTLD patient tissue.

As functional consequences of pathologies are difficult to ascertain in post-mortem patient brain, whether proteinaceous inclusions are toxic or whether they represent a protective mechanism by sequestering more toxic soluble protein species is often debated. Indeed, one study found that although nucleolar size is decreased in the brains of patients with Alzheimer’s disease compared with controls, it is increased in patients with asymptomatic Alzheimer’s disease, who exhibit Aβ plaques and neurofibrillary tangles but normal cognitive function [11], suggesting that increased nucleolar volume could also represent an early neuroprotective mechanism.

Surprisingly, we also uncovered a third association, between *C9orf72* repeat RNA pathology and nucleolar volume, albeit an association with a smaller effect size (1.2-fold increase) than the change from the presence of poly(GR) inclusions (1.8-fold increase). This nucleolar enlargement was predominantly mediated by direct association of RNA foci with the nucleolar structure, evidenced by a larger increase in volume in nucleoli that colocalised with foci. *C9orf72* sense repeat RNA has previously been shown to be able to interact with the nucleolar proteins in vitro [5, 10]. Depletion of RNA-binding proteins by RNA foci is hypothesised to be a key pathomechanism in other non-coding repeat expansion disorders, which are characterised by certain RNA-binding proteins being found within RNA foci. However, the localisation pattern of RNA foci and nucleophosmin that we detected did not recapitulate these findings, as RNA foci were generally detected on the edge of nucleolar structures.

Rather than nucleolar enlargement representing an increase in cell metabolism, it might infer a dispersal of the normal physiological structure of the organelle. Membraneless structures such as nucleoli are dynamic and respond to environmental conditions and cell stress, consequently *C9orf72* arginine-rich DPR proteins or repeat RNA could either disrupt or prevent reformation of these structures. Indeed, recent studies that examined the arginine-rich DPR proteins interacting with LCD-containing proteins showed perturbation of phase separation, a process thought to recapitulate the formation of membraneless organelles [14, 15]. In addition, the arginine-rich DPRs themlseves can phase separate and alter the phase separation dynamics of other proteins [3]. Alterations in membraneless organelles other than the nucleolus have also been observed in studies of C9FTD/ALS. Overexpression of *C9orf72* repeats in cells causes an increase in the percentage of cells containing stress granules [29]. In addition overexpressed poly(GR) or poly(PR) in cells can localise to [3, 35] and prevent the formation of stress granules subjected to a stressor [32]. In C9FTD/ALS patient cells repeat RNA is localised to FMRP-positive transport granules [4], and both the number of stress granules [6] and P bodies [10] are increased. In the case of repeat RNA, nucleolar disruption might occur by sequestration of nucleolar proteins by soluble RNA species which would not be detected using the methodology in this study. The presence of RNA foci could reflect cell nuclei in which the concentration of retained repeat RNA is the highest, and thus a nuclear environment in which this scenario is more likely to occur.

In addition to effect size, the proportion of the C9FTLD neuronal populations affected by the bidirectional nucleolar changes that we have observed are also important to consider. Overall, in C9FTLD frontal cortical neurons a 25% reduction in nucleolar size exists compared with controls. However, nucleolar size within these neurons is clearly heterogeneous. The third of neurons that contain RNA foci show a 1.2-fold nucleolar enlargement, and the 6% of neurons that contain poly(GR) inclusions show a 1.8-fold enlargement, compared with neurons without these pathologies. While we can speculate on how this change affects neuronal function within these subsets of neurons, how functionality of the frontal cortex is affected at a network level is unknown.

## Conclusions

In conclusion, we have provided the first evidence for nucleolar stress in C9FTLD patient brain, by using three-dimensional volumetric imaging. Bidirectional changes in nucleolar volume dependent on the presence or absence of *C9orf72* repeat RNA or protein pathologies show the heterogeneity of pathomechanisms in patient neurons, but support findings in current experimental models and have important implications for understanding the complex disease processes involved in C9FTLD/ALS.

## Additional file

Additional file 1: 
**Table S1** and **Figures S1-S7.** **Figure S1.** No difference in number of nucleophosmin-positive nucleoli or nuclear size of neurons in C9FTLD patient brain compared with neurons from non-neurodegenerative disease control brain. **a** Quantification of the number of nucleophosmin (NPM)-positive nucleolar structures per neuron in frontal cortex from C9FTLD patient brain (orange) and controls (blue). Bars shown represent average and SEM of heterozygous cases. **b** Quantification of neuronal nuclear volume determined by DAPI staining (in nucleophosmin-immunostained cases, Fig. 1). Median nuclear volume was no different between neurons from C9FTLD cases and controls. Each dot represents an individual case with the homozygous C9FTLD case shown in red and the average and SEM of heterozygous cases shown as long and short horizontal bars, respectively. Significance was determined by unpaired *t* test: ns = non-significant. **c**,**d** Correlation of nucleophosmin and nuclear (DAPI) volumes per individual neuron in controls (**c**) and C9FTLD patient brain (**d**). Nucleophosmin volume in controls and C9FTLD cases was positively correlated with nuclear volume (*p* < 0.0001, both), but with a very low fit (R^2^ = 0.039 and 0.043 respectively), therefore nucleolar volumes were not corrected for nuclear volume. Each dot represents values from an individual neuron, linear regression in red. **Figure S2.** No difference in nucleolin volume between neurons from C9FTLD patient brain and neurologically-normal controls. **a** Representative images of frontal cortex from neurologically-normal controls and heterozygous (C9 Het) and homozygous (C9 Hom) C9FTLD cases immunostained for the nucleolar protein nucleolin (NCL, *green*), the neuronal marker (NeuN, *magenta*) with DAPI nuclear stain (*blue*). Scale bar represents 2 μm. **b** Quantification of the number of nucleolin-positive nucleolar structures per neuron in frontal cortex from C9FTLD patient brain (orange) and controls (blue). Bars shown represent average and SEM of heterozygous cases. **c**,**d** Quantification of neuronal nucleolar volume determined by nucleolin immunoreactivity. Frequency distribution of pooled control and C9FTLD (heterozygous cases only) nucleolin volumes were similar (**c**) and median nucleolin volume was no different in neurons from C9FTLD cases and controls (**d**). **e** Quantification of neuronal nuclear volume determined by DAPI staining. Median nuclear volume was no different in neurons from C9FTLD cases and controls. In **d** and **e**, each dot represents an individual case with the homozygous C9FTLD case shown in red and the average and SEM of heterozygous cases shown as long and short horizontal bars, respectively. Significance was determined by unpaired *t* test: ns = non-significant. **f**,**g** Correlation of nucleolin and nuclear (DAPI) volumes per individual neuron in controls (**f**) and C9FTLD patient brain (**g**). Nucleolin volume in controls and C9FTLD cases was positively correlated with nuclear volume (*p* < 0.0001, both), but with a very low fit (R^2^ = 0.038 and 0.091 respectively). Each dot represents values from an individual neuron, linear regression in red. **Figure S3.** No difference in number of nucleophosmin-positive nucleoli or nuclear size of neurons in poly(GR) inclusion-bearing neurons in C9FTLD patient brain than in neurons without inclusions. **a** Quantification of the number of nucleophosmin (NPM)-positive nucleolar structures per neuron in frontal cortex from C9FTLD patient brain in neurons with (red, GR+) or without (orange, GR-) poly(GR) inclusions. Bars shown represent average and SEM of heterozygous cases. **b** Quantification of neuronal nuclear volume determined by DAPI staining (in nucleophosmin-immunostained cases, Fig. 2). Median nuclear volume in C9FTLD cases was no different in neurons with poly(GR) inclusions than in neurons without inclusions. Each dot represents an individual case with the homozygous C9FTLD case shown in red, grey lines link medians from the same cases in neurons with or without poly(GR) inclusions, and the average and SEM of heterozygous cases are shown as long and short horizontal bars, respectively. Significance was determined by unpaired *t* test: ns = non-significant. **Figure S4.** Increased nucleolin volume in poly(GR) inclusion-bearing neurons in C9FTLD patient brain. **a** Representative images of frontal cortex from a heterozygous C9FTLD case immunostained for the nucleolar protein nucleolin (NCL, green), poly(GR) protein (red), the neuronal marker (NeuN, magenta) with DAPI nuclear stain (blue); a typical poly(GR) inclusion is arrowed. Scale bar represents 2 μm. **b** Quantification of the number of nucleolin-positive nucleolar structures per neuron in frontal cortex from C9FTLD patient brain in neurons with (red, GR+) or without (orange, GR-) poly(GR) inclusions. Bars shown represent average and SEM of heterozygous cases. **c**,**d** Quantification of neuronal nucleolar volume determined by nucleolin immunoreactivity. Frequency distribution analyses of pooled C9FTLD (heterozygous cases only) nucleolin volumes show a shift to increased volume in neurons bearing poly(GR) inclusions than in neurons without inclusions (**c**). Median nucleolin volume in C9FTLD cases was significantly larger in neurons with poly(GR) inclusions than in neurons without inclusions (**d**). **e** Quantification of neuronal nuclear volume determined by DAPI staining (in nucleolin-immunostained cases). Median nuclear volume in C9FTLD cases was no different in neurons with poly(GR) inclusions than in neurons without inclusions. In **d** and **e**, each dot represents an individual case with the homozygous C9FTLD case shown in red, grey lines link medians from the same cases in neurons with or without poly(GR) inclusions, and the average and SEM of heterozygous cases shown as long and short horizontal bars, respectively. Significance was determined by unpaired *t* test: ****p* < 0.001, ns = non-significant. **Figure S5.** Frequency of poly(GR) and poly(GA) inclusions in *Drosophila* adult neurons. Quantification of the percentage of neurons in *Drosophila* brain either induced or uninduced with 200 μM RU486 for gene expression of GR(100) or GA(100) for 7 days using the elav-GeneSwitch (elavGS) driver (pictures shown in Fig. 3). In both GR(100) and GA(100) flies expression of the transgene led to approximately 7% of neurons bearing poly(GR) or poly(GA) inclusions, respectively, compared to less than 1.5% in uninduced flies. The inclusions found in flies where protein expression had not been induced are likely due to the known leaky expression of the elav-GeneSwitch driver [1]. Bars represent the average and SEM. **Figure S6.** Poly(GR) inclusion and RNA foci pathologies in C9FTLD patient brain are only occasionally found in the same neurons. **a** Representative images of frontal cortex from heterozygous C9FTLD cases immunostained for the nucleolar protein nucleophosmin (NPM, *green*), poly(GR) protein (*white*) with RNA fluorescent in situ hybridisation for sense RNA foci (*red*) and DAPI nuclear stain (*blue*); a neuron that contains an RNA focus but no poly(GR) inclusion, and a rare neuron that contains both a poly(GR) inclusion and an RNA focus (Foci + GR+) are highlighted with dotted boxes. Nucleophosmin immunostaining was detected in poly(GR) inclusions (hollow arrow) due to cross-reactivity of the secondary antibodies, and was excluded from analyses. Neurons with both pathologies were excluded from analysis in Fig. 3 in order to focus on the effects of RNA foci. Scale bar represents 2 μm. **b**,**c** Quantification of the frequency of poly(GR) protein and RNA foci pathologies. RNA foci are much more frequent than poly(GR) pathology overall (**b**) and in all cases individually (**c**). The two pathologies do occasionally overlap, but no more than would be expected by their relative frequencies. **d** Quantification of the number of nucleophosmin-positive nucleolar structures per neuron in frontal cortex from C9FTLD patient brain in neurons with (red, Foci+) or without (orange, Foci-) RNA foci. Bars shown represent average and SEM of heterozygous cases. **e** Quantification of neuronal nuclear volume determined by DAPI staining from 4 out of 6 of the same cases (cases 9,12,14,18) using a previously published dataset [Mizielinska et al., 2013]. Median nuclear volume in C9FTLD cases was no different in neurons with or without RNA foci. In **b** and **e**, each dot represents an individual case, and the average and SEM of heterozygous cases are shown as long and short horizontal bars, respectively. Significance was determined by paired *t* test: ns = non-significant. **Figure S7.** Nucleolar volume in C9FTLD patient brain is increased to a greater extent in neurons bearing RNA foci that are associated with the nucleolus than in neurons with foci in the nucleoplasm. **a** Representative images of frontal cortex from a heterozygous C9FTLD case immunostained for the nucleolar protein nucleophosmin (NPM, green) with RNA fluorescent in situ hybridisation for sense RNA foci (red) and DAPI nuclear stain (blue); panels show a neuron that contains an RNA focus associated with a nucleophosmin-positive nucleoli (upper), and a neuron with a focus in the nucleoplasm (lower). Scale bar represents 2 μm. **b** Quantification of the frequency of RNA foci associated with nucleophosmin-positive nucleolar staining as a percentage of total RNA foci. **c** Quantification of neuronal nucleolar volume determined by nucleophosmin immunoreactivity. Median nucleolar volume in C9FTLD cases was significantly larger in neurons with RNA foci associated with nucleolar staining (nucleolar foci) than in neurons with foci in the nucleoplasm (non-nucleolar foci). Individually 5 out of the 6 cases followed this trend. Each dot represents an individual heterozygous C9FTLD case, grey lines link medians from the same cases in neurons with RNA foci associated with either nucleolar staining or the nucleoplasm, and the average and SEM are shown as long and short horizontal bars, respectively. Significance was determined by paired regression analysis: ***P <* 0.01. (PDF 1494 kb)
