# Supplementary material for: Bidirectional nucleolar dysfunction in C9orf72 frontotemporal lobar degeneration
Source: Acta Neuropathol Commun. 2017 Apr 18;5:29. doi: 10.1186/s40478-017-0432-x (PMC5395972; doi:10.1186/s40478-017-0432-x)
Supplement: Additional file 1: — Table S1 and Figures S1-S7. Figure S1. No difference in number of nucleophosmin-positive nucleoli or nuclear size of neurons in C9FTLD patient brain compared with neurons from non-neurodegenerative disease control brain. a Quantification of the number of nucleophosmin (NPM)-positive nucleolar structures per neuron in frontal cortex from C9FTLD patient brain (orange) and controls (blue). Bars shown represent average and SEM of heterozygous cases. b Quantification of neuronal nuclear volume determined by DAPI staining (in nucleophosmin-immunostained cases, Fig. 1). Median nuclear volume was no different between neurons from C9FTLD cases and controls. Each dot represents an individual case with the homozygous C9FTLD case shown in red and the average and SEM of heterozygous cases shown as long and short horizontal bars, respectively. Significance was determined by unpaired t test: ns = non-significant. c,d Correlation of nucleophosmin and nuclear (DAPI) volumes per individual neuron in controls (c) and C9FTLD patient brain (d). Nucleophosmin volume in controls and C9FTLD cases was positively correlated with nuclear volume (p < 0.0001, both), but with a very low fit (R2 = 0.039 and 0.043 respectively), therefore nucleolar volumes were not corrected for nuclear volume. Each dot represents values from an individual neuron, linear regression in red. Figure S2. No difference in nucleolin volume between neurons from C9FTLD patient brain and neurologically-normal controls. a Representative images of frontal cortex from neurologically-normal controls and heterozygous (C9 Het) and homozygous (C9 Hom) C9FTLD cases immunostained for the nucleolar protein nucleolin (NCL, green), the neuronal marker (NeuN, magenta) with DAPI nuclear stain (blue). Scale bar represents 2 μm. b Quantification of the number of nucleolin-positive nucleolar structures per neuron in frontal cortex from C9FTLD patient brain (orange) and controls (blue). Bars shown represent average and SEM of heterozygous c [file 40478_2017_432_MOESM1_ESM.pdf]

Bidirectional nucleolar dysfunction in *C9orf72* patient brain with frontotemporal lobar degeneration

Acta Neuropathologica

Sarah Mizielinska, Charlotte E. Ridler, Rubika Balendra, Annora Thoeng, Nathan S. Woodling, Friedrich A. Grässer, Vincent Plagnol, Tammaryn Lashley, Linda Partridge, Adrian M. Isaacs

Corresponding author:
Dr Adrian Isaacs
Department of Neurodegenerative Disease, UCL Institute of Neurology, Queen Square, London WC1N 3BG
a.isaacs@ucl.ac.uk

Supplementary Table 1

Case number	Clinical diagnosis	Pathological diagnosis	Gender	Age at onset	Age at death	Duration	Inclusion in data set		
							Control vs. C9FTLD	C9FTLD GR±	C9FTLD Foci±
1	Neurologically normal	Normal control	M	N/A	69	N/A	y	y	
2	Neurologically normal	Normal control	F	N/A	73	N/A	y	y	
3	Neurologically normal	Normal control	M	N/A	88	N/A	y	y	
4	Neurologically normal	Normal control	F	N/A	83	N/A	y	y	
5	Neurologically normal	Normal control	F	N/A	80	N/A	y	y	
6	Neurologically normal	Normal control	F	N/A	86	N/A	y	y	
7	Neurologically normal	Normal control	F	N/A	93	N/A	y	y	
8	Neurologically normal	Normal control	F	N/A	91	N/A	y	y	
9	FTD	C9FTLD-TDP type A	M	62	68	6	y	y	y
10	FTD/MND	C9FTLD-TDP type A	M	66	71	5	y	y	y
11	FTD	C9FTLD-TDP type A	M	59	65	6	y	y	
12	FTD	C9FTLD-TDP type B	F	64	66	2	y	y	y
13	FTD (PNFA)	C9FTLD-TDP type A	F	57	62	5	y	y	
14	FTD	C9FTLD-TDP type A	M	54	60	6	y	y	y
15	FTD	C9FTLD-TDP type A	M	53	63	10	y	y	
16	FTD	C9FTLD-TDP type A	F	66	74	8	y	y	
17*	FTD	C9FTLD-TDP type A	M	43	45	2	y	y	
18	HD-like (early onset)	C9FTLD-TDP type A	F	56	67	11			y
19	FTD (bvFTD)	C9FTLD-TDP type A	F	58	66	8			y

C9orf72-associated frontotemporal lobar degeneration, C9FTLD; poly(glycine-arginine), GR; male, M; female, F; not applicable, N/A; yes, y; frontotemporal dementia, FTD; TAR DNA-binding protein-43, TDP; motor neuron disease, MND; progressive non-fluent aphasia, PNFA; Huntingdon's disease, HD; behavioural variant FTD, bvFTD; *Homozygous C9FTLD case.

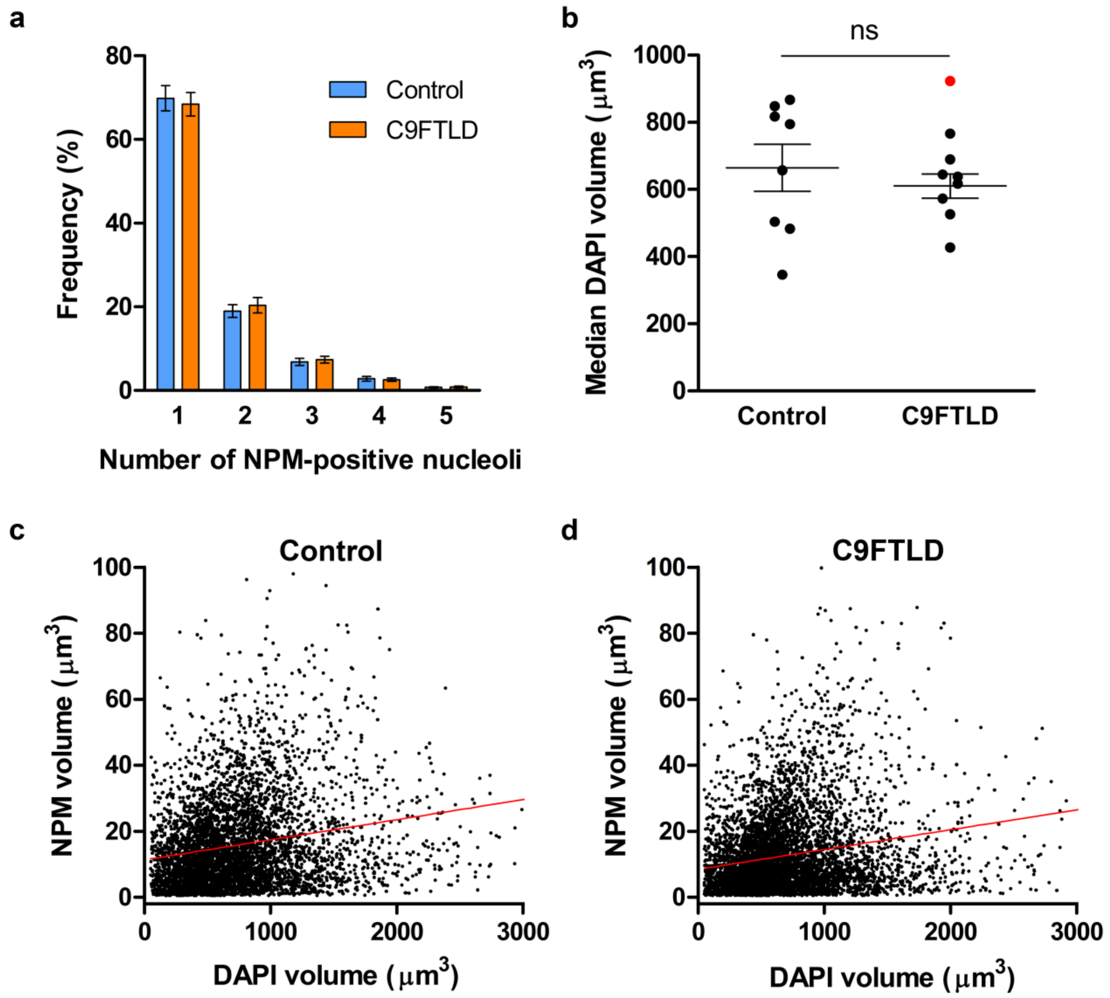

Supplementary Fig. 1 No difference in number of nucleophosmin-positive nucleoli or nuclear size of neurons in C9FTLD patient brain compared with neurons from non-neurodegenerative disease control brain. **a** Quantification of the number of nucleophosmin (NPM)-positive nucleolar structures per neuron in frontal cortex from C9FTLD patient brain (orange) and controls (blue). Bars shown represent average and SEM of heterozygous cases. **b** Quantification of neuronal nuclear volume determined by DAPI staining (in nucleophosmin-immunostained cases, Figure 1). Median nuclear volume was no different between neurons from C9FTLD cases and controls. Each dot represents an individual case with the homozygous C9FTLD case shown in red and the average and SEM of heterozygous cases shown as long and short horizontal bars, respectively. Significance was determined by unpaired t test: ns = non-significant. **c,d** Correlation of nucleophosmin and nuclear (DAPI) volumes per individual neuron in controls (**c**) and C9FTLD patient brain (**d**). Nucleophosmin volume in controls and C9FTLD cases was positively correlated with nuclear volume ($p < 0.0001$, both), but with a very low fit ($R^2 = 0.039$ and 0.043 respectively), therefore nucleolar volumes were not corrected for nuclear volume. Each dot represents values from an individual neuron, linear regression in red.

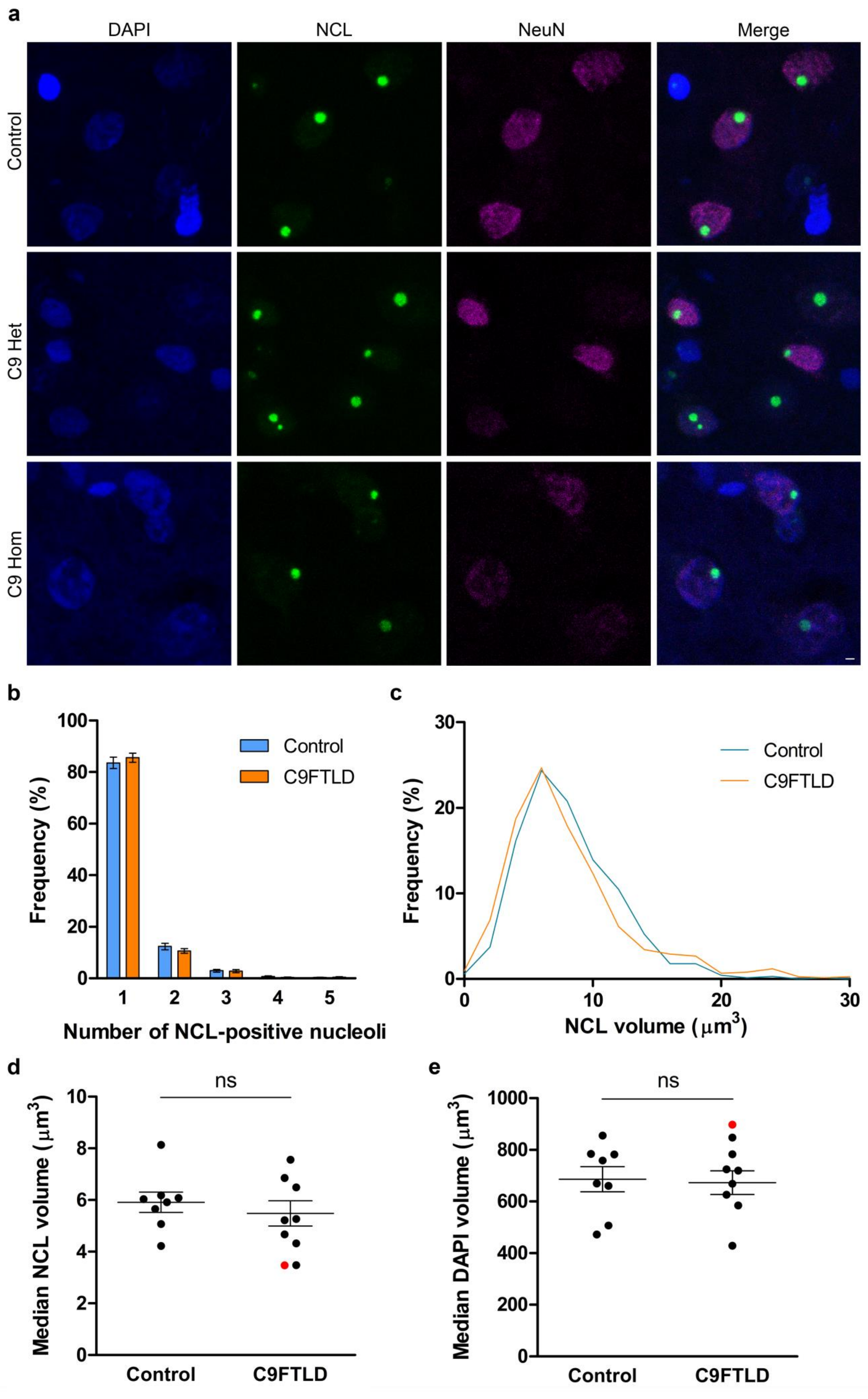
a
DAPI
NCL
NeuN
Merge
Control
C9 Het
C9 Hom
b
Frequency (%)
Control
C9FTLD
Number of NCL-positive nucleoli
c
Frequency (%)
Control
C9FTLD
NCL volume (μm^3)
d
ns
Median NCL volume (μm^3)
Control
C9FTLD
e
ns
Median DAPI volume (μm^3)
Control
C9FTLD

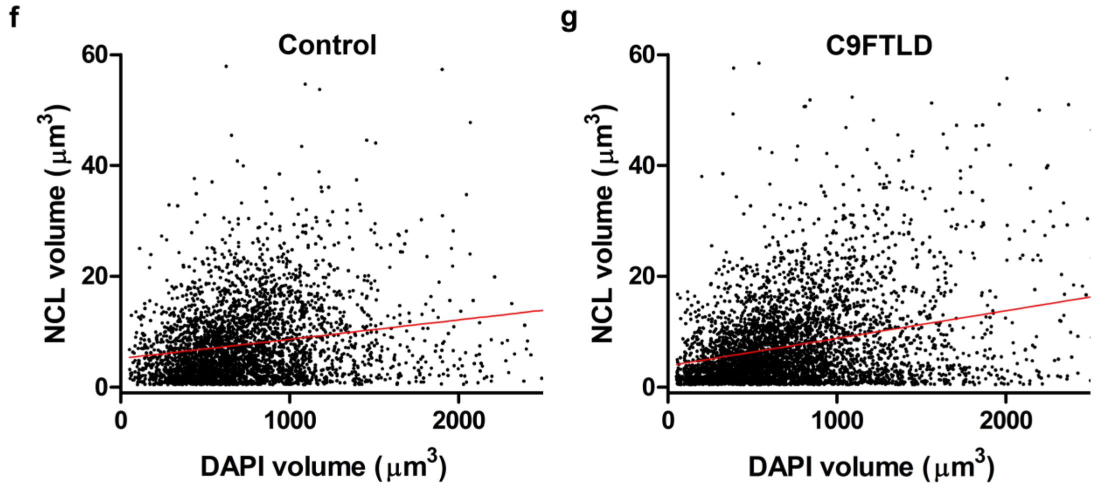

Supplementary Fig. 2 No difference in nucleolin volume between neurons from C9FTLD patient brain and neurologically-normal controls. **a** Representative images of frontal cortex from neurologically-normal controls and heterozygous (C9 Het) and homozygous (C9 Hom) C9FTLD cases immunostained for the nucleolar protein nucleolin (NCL, *green*), the neuronal marker (NeuN, *magenta*) with DAPI nuclear stain (*blue*). Scale bar represents 2 μm. **b** Quantification of the number of nucleolin-positive nucleolar structures per neuron in frontal cortex from C9FTLD patient brain (orange) and controls (blue). Bars shown represent average and SEM of heterozygous cases. **c,d** Quantification of neuronal nucleolar volume determined by nucleolin immunoreactivity. Frequency distribution of pooled control and C9FTLD (heterozygous cases only) nucleolin volumes were similar (**c**) and median nucleolin volume was no different in neurons from C9FTLD cases and controls (**d**). **e** Quantification of neuronal nuclear volume determined by DAPI staining. Median nuclear volume was no different in neurons from C9FTLD cases and controls. In **d** and **e**, each dot represents an individual case with the homozygous C9FTLD case shown in red and the average and SEM of heterozygous cases shown as long and short horizontal bars, respectively. Significance was determined by unpaired t test: ns = non-significant. **f,g** Correlation of nucleolin and nuclear (DAPI) volumes per individual neuron in controls (**f**) and C9FTLD patient brain (**g**). Nucleolin volume in controls and C9FTLD cases was positively correlated with nuclear volume ($p < 0.0001$, both), but with a very low fit ($R^2 = 0.038$ and 0.091 respectively). Each dot represents values from an individual neuron, linear regression in red.

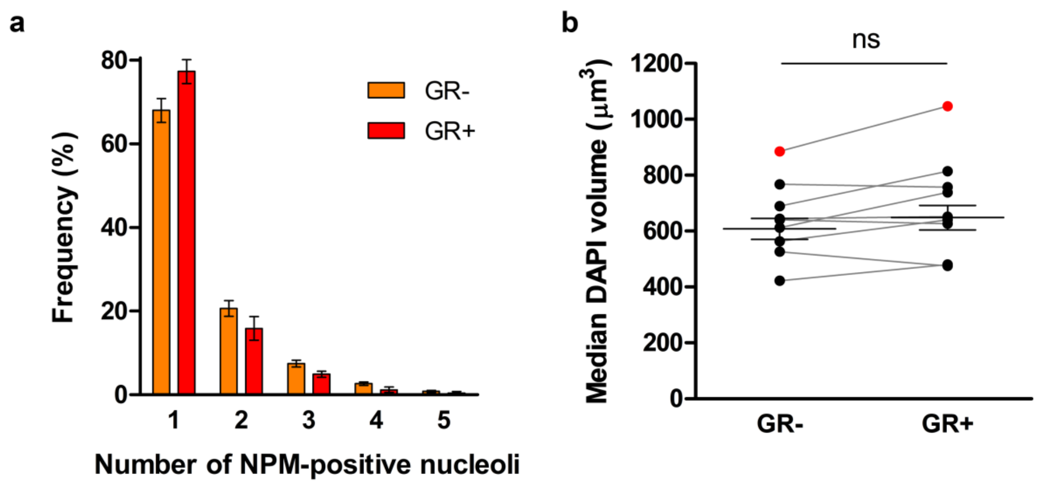

Supplementary Fig. 3 No difference in number of nucleophosmin-positive nucleoli or nuclear size of neurons in poly(GR) inclusion-bearing neurons in C9FTLD patient brain than in neurons without inclusions. **a** Quantification of the number of nucleophosmin (NPM)-positive nucleolar structures per neuron in frontal cortex from C9FTLD patient brain in neurons with (red, GR+) or without (orange, GR-) poly(GR) inclusions. Bars shown represent average and SEM of heterozygous cases. **b** Quantification of neuronal nuclear volume determined by DAPI staining (in nucleophosmin-immunostained cases, Figure 2). Median nuclear volume in C9FTLD cases was no different in neurons with poly(GR) inclusions than in neurons without inclusions. Each dot represents an individual case with the homozygous C9FTLD case shown in red, grey lines link medians from the same cases in neurons with or without poly(GR) inclusions, and the average and SEM of heterozygous cases are shown as long and short horizontal bars, respectively. Significance was determined by unpaired t test: ns = non-significant.

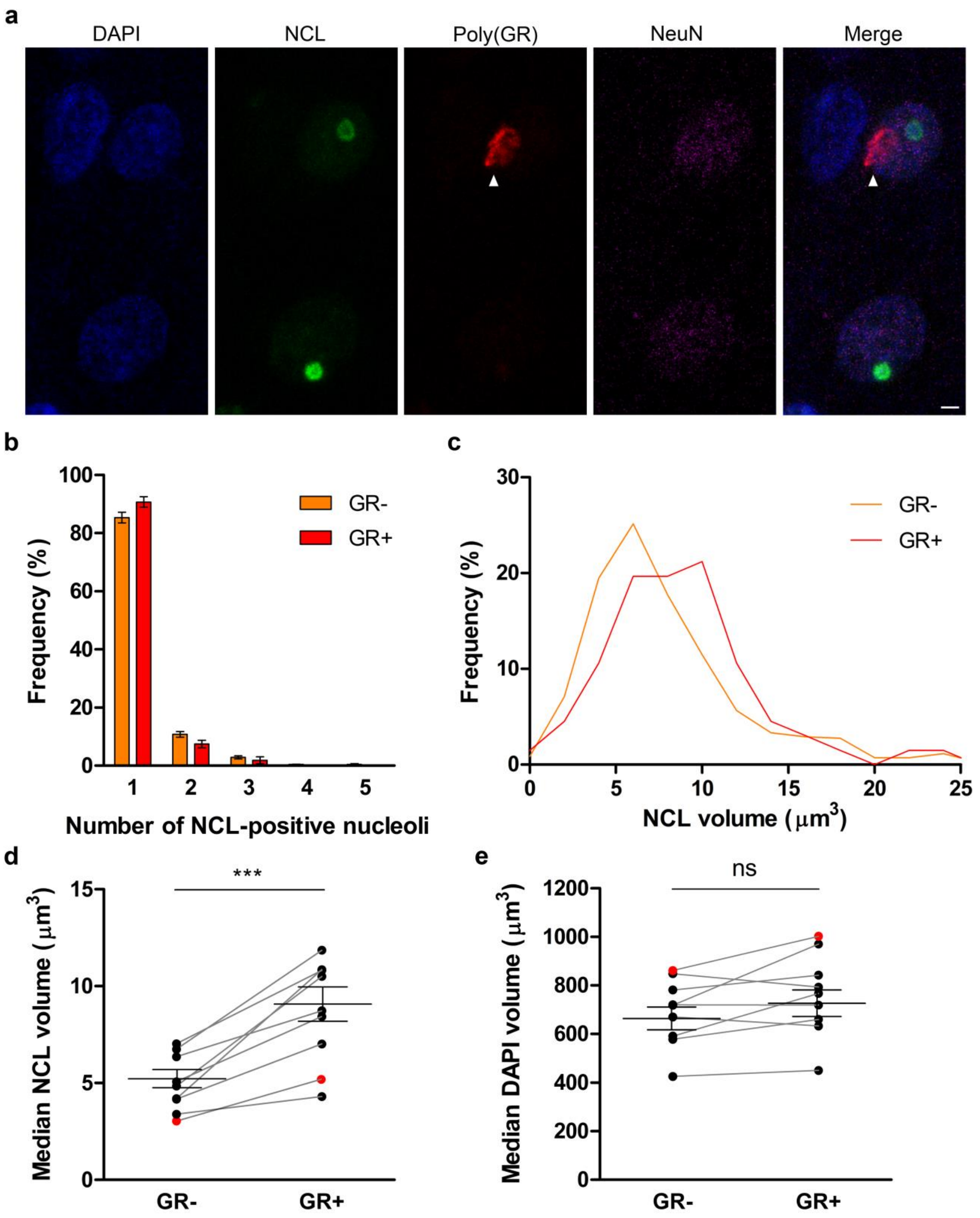

Supplementary Fig. 4 Increased nucleolin volume in poly(GR) inclusion-bearing neurons in C9FTLD patient brain. **a** Representative images of frontal cortex from a heterozygous C9FTLD case immunostained for the nucleolar protein nucleolin (NCL, green), poly(GR) protein (red), the neuronal marker (NeuN, magenta) with DAPI nuclear stain (blue); a typical poly(GR) inclusion is arrowed. Scale bar represents 2 μm. **b** Quantification of the number of nucleolin-positive nucleolar structures per neuron in frontal cortex from C9FTLD patient brain in neurons with (red, GR+) or without (orange, GR-) poly(GR) inclusions. Bars shown represent average and SEM of heterozygous cases. **c**,**d** Quantification of neuronal nucleolar volume determined by nucleolin immunoreactivity. Frequency

distribution analyses of pooled C9FTLD (heterozygous cases only) nucleolin volumes show a shift to increased volume in neurons bearing poly(GR) inclusions than in neurons without inclusions (**c**). Median nucleolin volume in C9FTLD cases was significantly larger in neurons with poly(GR) inclusions than in neurons without inclusions (**d**). **e** Quantification of neuronal nuclear volume determined by DAPI staining (in nucleolin-immunostained cases). Median nuclear volume in C9FTLD cases was no different in neurons with poly(GR) inclusions than in neurons without inclusions. In **d** and **e**, each dot represents an individual case with the homozygous C9FTLD case shown in red, grey lines link medians from the same cases in neurons with or without poly(GR) inclusions, and the average and SEM of heterozygous cases shown as long and short horizontal bars, respectively. Significance was determined by unpaired t test: ***p < 0.001, ns = non-significant.

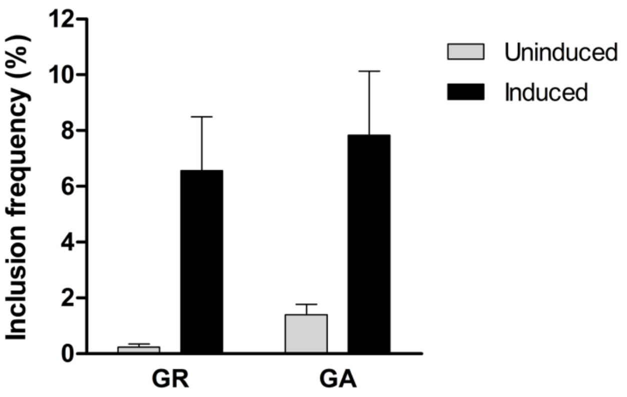

Supplementary Fig. 5 Frequency of poly(GR) and poly(GA) inclusions in *Drosophila* adult neurons. Quantification of the percentage of neurons in *Drosophila* brain either induced or uninduced with 200 µM RU486 for gene expression of GR(100) or GA(100) for 7 days using the elav-GeneSwitch (elavGS) driver (pictures shown in Figure 3). In both GR(100) and GA(100) flies expression of the transgene led to approximately 7% of neurons bearing poly(GR) or poly(GA) inclusions, respectively, compared to less than 1.5% in uninduced flies. The inclusions found in flies where protein expression had not been induced are likely due to the known leaky expression of the elav-GeneSwitch driver [2]. Bars represent the average and SEM.

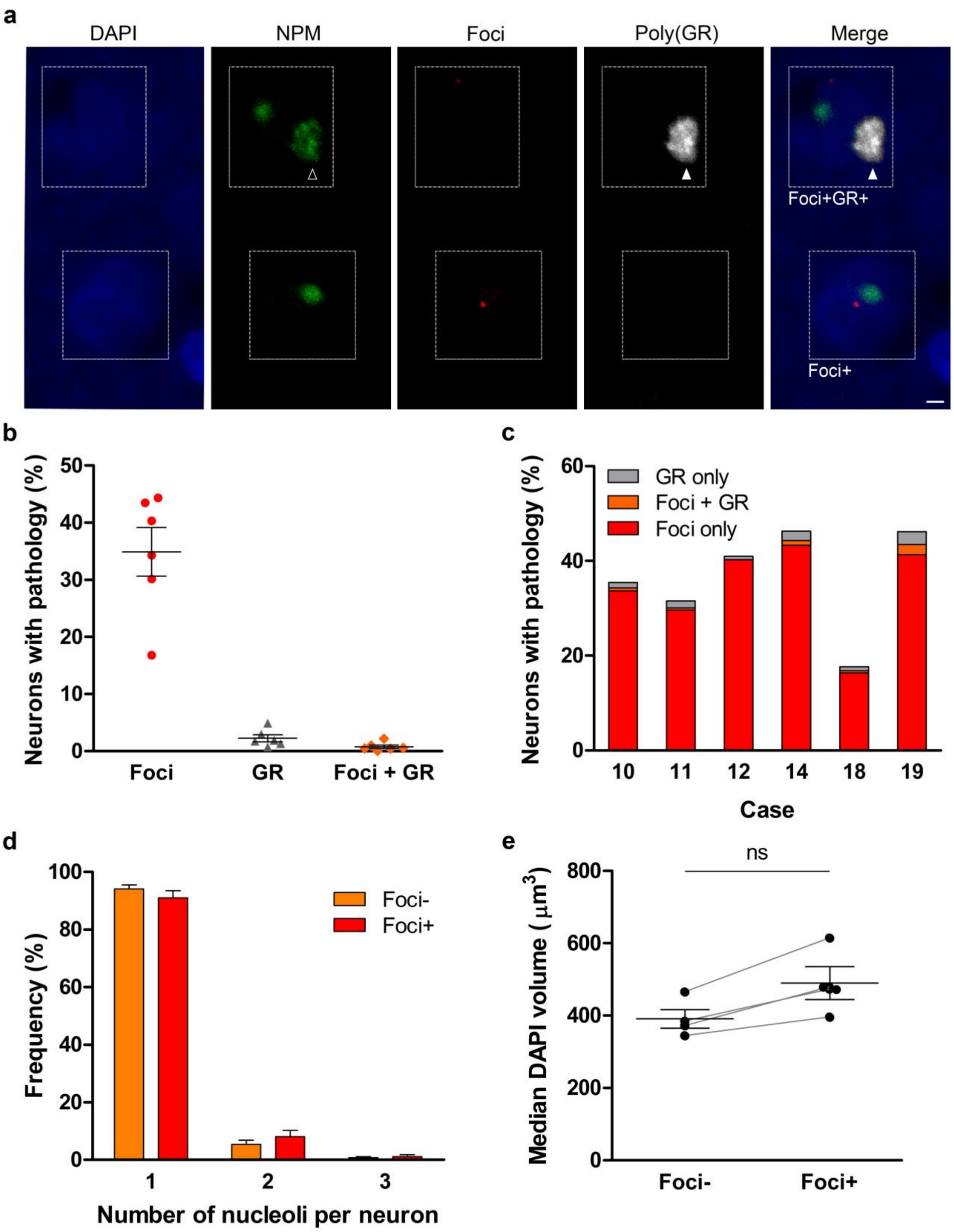

Supplementary Fig. 6 Poly(GR) inclusion and RNA foci pathologies in C9FTLD patient brain are only occasionally found in the same neurons. **a** Representative images of frontal cortex from heterozygous C9FTLD cases immunostained for the nucleolar protein nucleophosmin (NPM, *green*), poly(GR) protein (*white*) with RNA fluorescent in situ hybridisation for sense RNA foci (*red*) and DAPI nuclear stain (*blue*); a neuron that contains an RNA focus but no poly(GR) inclusion, and a rare neuron that contains both a poly(GR) inclusion and an RNA focus (Foci+GR+) are highlighted with dotted boxes. Nucleophosmin immunostaining was detected in poly(GR) inclusions (hollow arrow) due to cross-reactivity of the secondary antibodies, and was excluded from analyses. Neurons with

both pathologies were excluded from analysis in Figure 3 in order to focus on the effects of RNA foci. Scale bar represents 2 µm. **b,c** Quantification of the frequency of poly(GR) protein and RNA foci pathologies. RNA foci are much more frequent than poly(GR) pathology overall (**b**) and in all cases individually (**c**). The two pathologies do occasionally overlap, but no more than would be expected by their relative frequencies. **d** Quantification of the number of nucleophosmin-positive nucleolar structures per neuron in frontal cortex from C9FTLD patient brain in neurons with (red, Foci+) or without (orange, Foci-) RNA foci. Bars shown represent average and SEM of heterozygous cases. **e** Quantification of neuronal nuclear volume determined by DAPI staining from 4 out of 6 of the same cases (cases 9,12,14,18) using a previously published dataset [1]. Median nuclear volume in C9FTLD cases was no different in neurons with or without RNA foci. In **b** and **e**, each dot represents an individual case, and the average and SEM of heterozygous cases are shown as long and short horizontal bars, respectively. Significance was determined by paired t test: ns = non-significant.

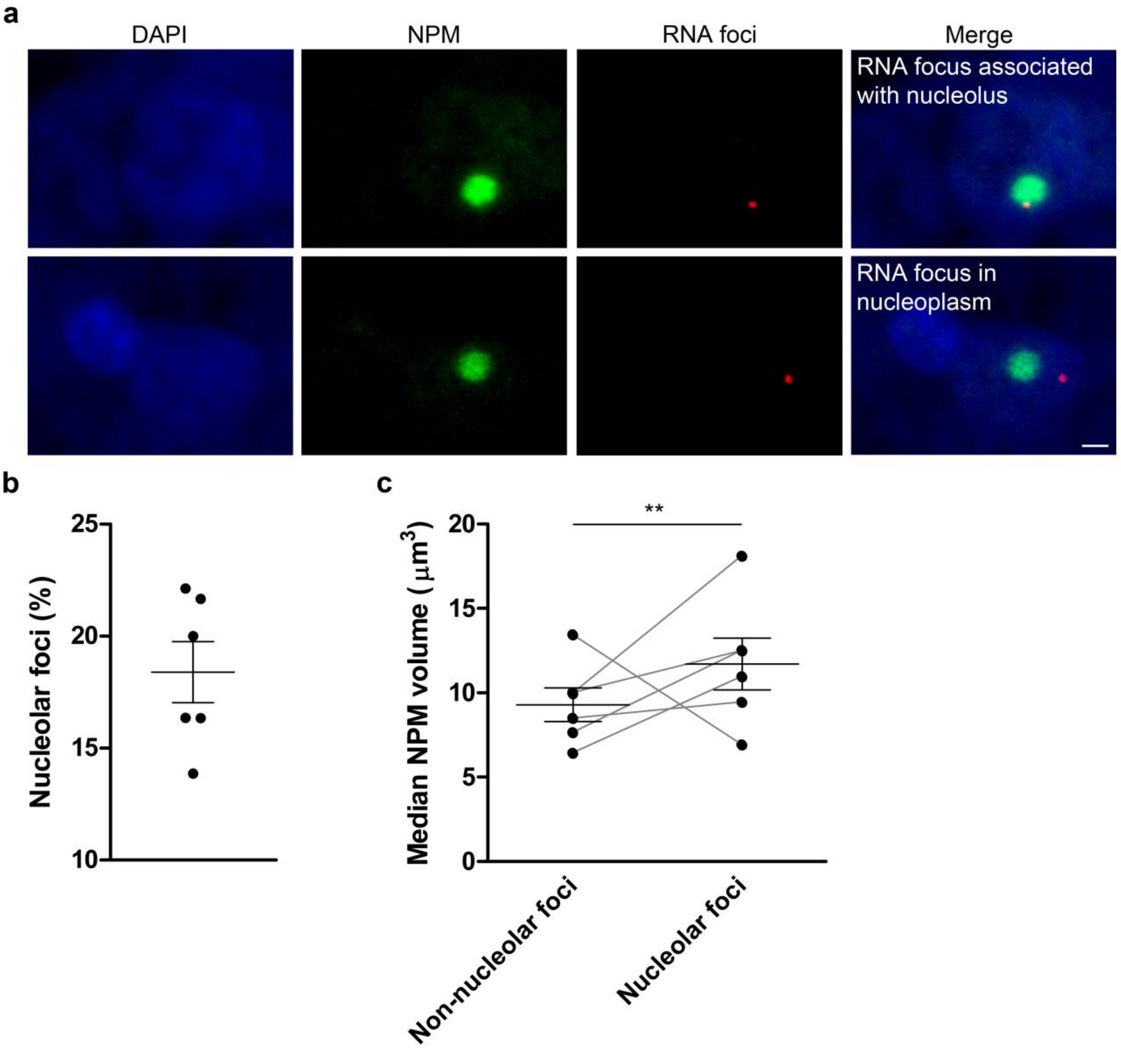

Supplementary Fig. 7 Nucleolar volume in C9FTLD patient brain is increased to a greater extent in neurons bearing RNA foci that are associated with the nucleolus than in neurons with foci in the nucleoplasm. **a** Representative images of frontal cortex from a heterozygous C9FTLD case immunostained for the nucleolar protein nucleophosmin (NPM, *green*) with RNA fluorescent in situ hybridisation for sense RNA foci (*red*) and DAPI nuclear stain (*blue*); panels show a neuron that contains an RNA focus associated with a nucleophosmin-positive nucleoli (upper), and a neuron with a focus in the nucleoplasm (lower). Scale bar represents 2 µm. **b** Quantification of the frequency of RNA foci associated with nucleophosmin-positive nucleolar staining as a percentage of total RNA foci. **c** Quantification of neuronal nucleolar volume determined by nucleophosmin immunoreactivity. Median nucleolar volume in C9FTLD cases was significantly larger in neurons with RNA foci associated with nucleolar staining (nucleolar foci) than in neurons with foci in the nucleoplasm (non-nucleolar foci). Individually 5 out of the 6 cases followed this trend. Each dot represents an individual heterozygous C9FTLD case, grey lines link medians from the same cases in neurons with RNA foci associated with either nucleolar staining or the nucleoplasm, and the average and SEM are shown as long and short horizontal bars, respectively. Significance was determined by paired regression analysis: $^{**}p < 0.01$.

References

1. Mizielinska S, Lashley T, Norona FE, Clayton EL, Ridler CE, Fratta P, Isaacs AM (2013) C9orf72 frontotemporal lobar degeneration is characterised by frequent neuronal sense and antisense RNA foci. Acta Neuropathol 126:845-857.

2. Poirier L, Shane A, Zheng J, Seroude L (2008) Characterization of the Drosophila gene-switch system in aging studies: a cautionary tale. Aging Cell 7:758-770.
